# Supplementary material for: Clinical Utility of the Tokyo Guidelines 2018 for Acute Cholangitis in the Emergency Department and Comparison with Novel Markers (Neutrophil-to-Lymphocyte and Blood Nitrogen Urea-to-Albumin Ratios)
Source: J Clin Med. 2024 Apr 16;13(8):2306. doi: 10.3390/jcm13082306 (PMC11051285; doi:10.3390/jcm13082306)
Supplement: Supplementary file 1 [file jcm-13-02306-s001.zip › jcm-2927086-supplementary.pdf]

Supplement Table S1. Comparison of ERCP status in patients according to BAR, NLR, CRP, and TG2018.

Factor	***ERCP***		***p***
	No (n=155)	*Yes (n=325)*	
BAR	*4.09 (1.12-111.11)*	*3.73 (1.00-24.37)*	*0.006*
NLR	*10.04 (0.95-194.20)*	*9.59 (0.67-88.45)*	*0.866*
CRP	*4.72 (0.04-26.15)*	*2.92 (0.03-31.02)*	*0.035*
TG2018	*2 (1-3)*	*1 (1-3)*	*0.102*

Abbreviations: ERCP; Endoscopic retrograde choangiopancreatography, BAR; blood urea nitrogen to albumin ratio, NLR; neutrophil to lymphocyte ratio, CRP; c-reactive protein, TG2018; tokyo guideline 2018,.

Supplement Table S2. Comparison of clinical outcomes according to TG2018 grade.

	Grade 1 (n=254)	*Grade 2 (n=120)*	*Grade 3 (n=108)*	*P*
Intensive care	*8 (3.1%)*	*12 (10.0%)*	*61 (56.5%)*	*0.000*
Long-term hospital stays	*33 (13.0%)*	*30 (25.0%)*	*48 (44.4%)*	*0.000*
PTBD	*17 (6.7%)*	*9 (7.5%)*	*23 (21.3%)*	*0.000*
Endotracheal intubation	*1 (0.4%)*	*0*	*13 (12.0%)*	*0.000*

Abbreviations: PTBD; percutaneous transhepatic bile drainage

Supplement Table S3. Comparison of BAR, NLR, CRP according to TG2018 grade.

	Grade 1 (n=254)	*Grade 2 (n=120)*	*Grade 3 (n=108)*	*P*
BAR	*3.24 (1.00-24.37)*	*4.33 (1.47-23.82)*	*6.34 (1.23-111.11)*	*0.000*
NLR	*6.74 (0.76-79.92)*	*12.74 (0.67-81.33)*	*15.66 (0.81-194.20)*	*0.000*
CRP	*1.16 (0.03-26.15)*	*5.61 (0.11-30.14)*	*7.53 (0.03-31.02)*	*0.000*

Abbreviations: BAR; blood urea nitrogen to albumin ratio, NLR; neutrophil to lymphocyte ratio, CRP; c-reactive protein,
